# An integrated analysis of hyponatremia in cancer patients receiving platinum-based or nonplatinum-based chemotherapy in clinical trials (JCOG1405-A)

**DOI:** 10.18632/oncotarget.23536

**Published:** 2017-12-20

**Authors:** Yasumasa Ezoe, Junki Mizusawa, Hiroshi Katayama, Kozo Kataoka, Manabu Muto

**Affiliations:** ^1^ Department of Therapeutic Oncology, Kyoto University Graduate School of Medicine, Shogoin, Sakyo-ku, Kyoto 606-8507, Japan; ^2^ Japan Clinical Oncology Group (JCOG) Data Center/Operations Office, National Cancer Center Hospital, Tsukiji, Chuo-ku, Tokyo 104-0045, Japan

**Keywords:** hyponatremia, platinum agent, cisplatin, carboplatin, risk factors

## Abstract

**Background:**

Hyponatremia is a common electrolyte abnormality in cancer patients who receive chemotherapy. Among anticancer agents, platinum-based agents are reported to cause chemotherapy-induced hyponatremia. However, the actual incidence and risk factors remain unknown.

**Results:**

The reports of 29 trials were analyzed. The incidence of grade 3/4 hyponatremia was 11.9% in patients treated with platinum-based chemotherapy and 3.8% in those treated with nonplatinum-based regimens (*P* < 0.01). Univariable analysis revealed a high incidence of hyponatremia in patients receiving cisplatin, three-drug combination regimen, two-drug combination regimen with amrubicin or irinotecan, or high-dose cisplatin (weekly equivalent cisplatin dose ≥20 mg/m^2^), and in patients with small-cell lung cancer.

**Conclusion:**

This is the first report of the actual incidence and the potential risk factors of chemotherapy-induced hyponatremia. Careful monitoring of serum sodium level is needed when platinum-based chemotherapy is administered.

**Methods:**

This study included all clinical trials of systemic chemotherapies for solid cancers that were conducted by the Japan Clinical Oncology Group (JCOG) after January 2000 and of which the patient enrolment was completed by January 2014. The latest reports of each trial were used for analysis. The incidence of chemotherapy-induced grade 3/4 hyponatremia and the potential risk factors were investigated with univariable analysis.

## INTRODUCTION

Hyponatremia is a common electrolyte disorder in cancer patients. However, recent studies suggest that hyponatremia might be a negative prognostic factor for cancer patients therefore its early detection and appropriate management might improve patient outcome [1–3]. The reported incidence of hyponatremia in cancer patients varies greatly according to the cancer type, clinical setting, and the serum sodium cutoff point, from 4% to 44% [4–7]. Hyponatremia in cancer patients can be caused by a number of factors including gastrointestinal losses, cardiac failure, diabetes insipidus, cancer-induced physiological changes or hormonal secretion, pulmonary diseases, central nervous system disorders, several drugs other than anticancer treatments (e.g., chlorpropamide, selective serotonin reuptake inhibitors, carbamazepine, antipsychotics, and vasopressin analogues), and several anticancer treatments [8, 9].

In clinical practice, chemotherapy-induced hyponatremia is an important adverse event because a rapid decrease in serum sodium level leads to disturbance of consciousness, convulsions, respiratory arrest, and treatment-related death in the worst-case scenario. In the event of chemotherapy-induced hyponatremia, chemotherapy may be discontinued or a dose reduction could be indicated. Currently, no effective prevention methods have been found; therefore, the only effective approach to avoid serious hyponatremia is early detection and appropriate treatment [7].

Among several anticancer agents, vinca alkaloids, alkylating agents, and platinum compounds are reported to be the chemotherapeutic agents that cause hyponatremia [7, 10]. Of these, platinum compounds are positioned as one of the key drugs used to treat major solid cancers, and are therefore frequently used in many chemotherapy regimens. As a result, platinum-induced hyponatremia is experienced more frequently than that associated with other chemotherapeutic agents in clinical practice. However, most of the previous reports were case studies and the exact incidence and risk factors of platinum-induced hyponatremia remain uncertain [11].

In this study, to evaluate the actual incidence and the potential risk factors of platinum-induced hyponatremia, we performed an integrated analysis of hyponatremia in patients with solid cancers receiving platinum-based or nonplatinum-based chemotherapy using the data from completed clinical trials conducted by the Japan Clinical Oncology Group (JCOG) [12–39].

## RESULTS

### Study selection

**Figure 1:**
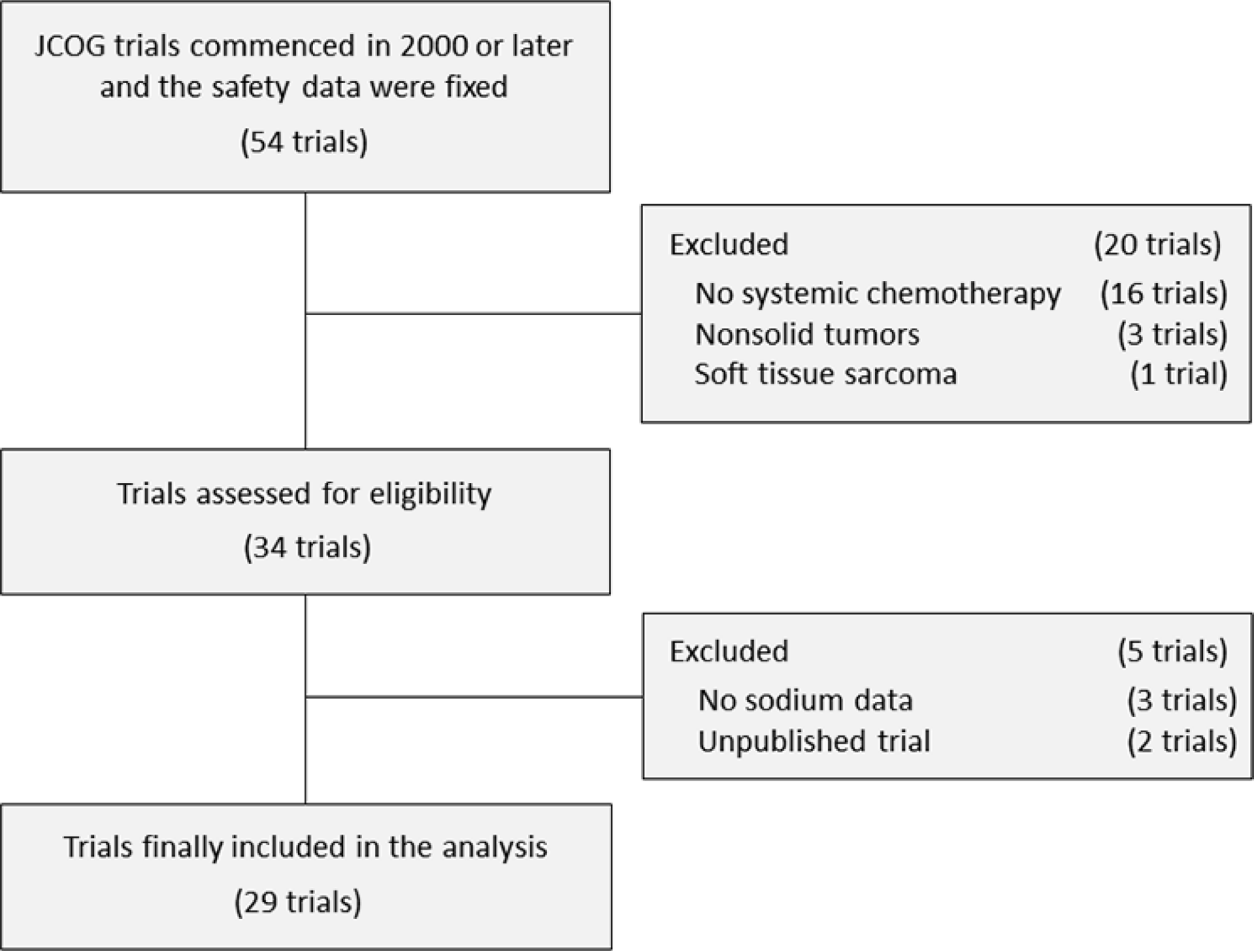
Selection process for the JCOG trials

We identified 54 potentially relevant trials. All study protocols were screened, and 25 trials were excluded. The selection process and reasons for exclusion are detailed in Figure 1. A total of 29 trials were included in the final analysis.

### Study, patients, and treatment characteristics

The reports of 29 phase II and III trials including 44 treatment arms (platinum-based chemotherapy, 27 treatment arms; nonplatinum-based chemotherapy, 17 treatment arms) were analyzed. The characteristics of each trial are summarized in Table 1A and 1B. The included trials consisted of three phase I/II trials (only the data of the phase II section were evaluated in this study), 10 phase II trials, one feasibility study, and 15 phase III trials. The target malignancies of included trials were non-small-cell lung cancer (6 trials), esophageal cancer (5), gastric cancer (5), small-cell lung cancer (3), ovarian, fallopian tube and peritoneal cancer (3), cervical cancer (2), colorectal cancer (1), pancreatic cancer (1), breast cancer (1), head and neck cancer (1), and bladder cancer (1).

**Table 1A:** Characteristics of JCOG clinical trials included in this study (Platinum-based chemotherapy arms)

JCOG trial No.	Phase	Malignancy	No. of patients	Regimen	Platinum administration schedule	Cisplatin administration method	Cisplatin dosage (DI, mg/m^2^/week)	Median age (years)	Reference
0001	II	Gastric	55	Cisplatin and irinotecan	80 mg/m^2^ on day 1 every 4 weeks for 8 or 12 weeks	bolus	high (20)	63	12
0102	III	Cervical	66	Cisplatin, bleomycin, mitomycin and vincristine	14 mg/m^2^ on days 1–5 every 3 weeks for 6 weeks	split	high (23.3)	47	13
0204	rII	Non-small-cell lung	40	Cisplatin and docetaxel	80 mg/m^2^ on day 1 every 4 weeks for 8 weeks	bolus	high (20)	62.5	19
0206	FS	Ovarian, fallopian tube, peritoneal	53	Carboplatin and paclitaxel (neoadjuvant)	AUC 6 on day 1 every 3 weeks for 12 weeks	-	-	55	21
			46	Carboplatin and paclitaxel (adjuvant)	AUC 6 on day 1 every 3 weeks for 12 weeks	-	-		
0207	III	Non-small-cell lung	63	Cisplatin and docetaxel	25 mg/m^2^ on days 1, 8 and 15 every 4 weeks	split	low (18.8)	76	22
0209	III	Bladder	56	Cisplatin, methotrexate, vinblastine and Adriamycin	70 mg/m^2^ on day 2 every 4 weeks for 8 weeks	bolus	low (17.5)	63	23
0210	II	Gastric	49	Cisplatin and S1	60 mg/m^2^ on day 8 every 4 weeks for 8 weeks	bolus	low (15)	61	24
0301	III	Non-small-cell lung	96	Carboplatin	30 mg/m^2^ on days 1-5 weekly for 4 weeks	-	-	77	25
0303	III	Esophageal	70	Cisplatin and fluorouracil	70 mg/m^2^ on day 1 every 4 weeks for 8 weeks	bolus	low (17.5)	63	26
			70	Cisplatin and fluorouracil	4 mg/m^2^ on days 1–5 weekly for 6 weeks	split	high (20)	62	
0402	I/II^*^	Non-small-cell lung	38	Cisplatin and vinorelbine	80 mg/m^2^ on day 1 every 3 weeks for 6 weeks	bolus	high (26.7)	59.5	27
0405	II	Gastric	52	Cisplatin and S1	60 mg/m^2^ on day 8 every 4 weeks for 8 or 12 weeks	bolus	how (15)	63	28
0502	III	Esophageal	165	Cisplatin and fluorouracil	70 mg/m^2^ on day 1 every 4 weeks for 8 weeks	bolus	low (17.5)	B; 69 D; 65	unpublished
0505	III	Cervical	125	Cisplatin and paclitaxel	50 mg/m^2^ on day 2 every 3 weeks for 18 weeks	bolus	low (16.7)	53	31
			126	Carboplatin and paclitaxel	AUC 5 on day 1 every 3 weeks for 18 weeks	-	-	53	
0508	II	Esophageal	96	Cisplatin and fluorouracil	70 mg/m^2^ on day 1 every 4 weeks for 8 weeks	bolus	low (17.5)	63	unpublished
0509	III	Small-cell lung	142	Cisplatin and irinotecan	60 mg/m^2^ on day 1 every 4 weeks for 16 weeks	bolus	low (15)	63	33
			140	Cisplatin and amrubicin	60 mg/m^2^ on day 1 every 3 weeks for 12 weeks	bolus	high (20)	63	
0602	III	Ovarian, fallopian tube, peritoneal	137	Carboplatin and paclitaxel (adjuvant)	AUC 6 on day 1 every 3 weeks for 24 weeks (adjuvant)	-	-	59	unpublished
			149	Carboplatin and paclitaxel (neoadjuvant plus adjuvant)	AUC 6 on day 1 every 3 weeks for 12 weeks (neoadjuvant) plus 12 weeks (adjuvant)	-	-	60.5	
0604	I/II^*^	Esophageal	38	Cisplatin and S1	75 mg/m^2^ on day 1 every 4 weeks for 8 weeks	bolus	low (18.8)	62	34
0605	III	Small-cell lung	90	Cisplatin, etoposide, and irinotecan	25 mg/m^2^ on days 1, 8 every 2 weeks for 10 weeks	split	high (25)	64	35
0706	II	Head and neck	45	Cisplatin and S1	20 mg/m^2^ on days 8–11 every 5 weeks for 10 weeks	split	low (16)	63	36
			40	Cisplatin and S1	20 mg/m^2^ on days 8–11 every 4 weeks for 8 weeks	split	high (20)		
0803	III	Non-small-cell lung	137	Cisplatin and docetaxel	25 mg/m^2^ on days 1, 8 and 15 every 4 weeks	split	low (18.8)	76	37
0807	I/II^*^	Esophageal	55	Cisplatin, fluorouracil, and docetaxel	80 mg/m^2^ on day 1 every 4 weeks	bolus	high (20)	61	38

Abbreviations: rII, randomized phase II; FS, feasibility study; AUC, area under the blood concentration time curve; I/II; phase I/II; DI, dose intensity.

^*^Data from phase II were used in this study.

**Table 1B:** Characteristics of JCOG clinical trials included in this study (Nonplatinum-based chemotherapy arms)

JCOG trial No.	Phase	Malignancy	No. of patients	Regimen	Median age (years)	Reference
0104	III	Non-small-cell lung	64	Docetaxel	62	14
			65	Docetaxel and gemcitabine	60	
0106	III	Gastric	117	Fluorouracil	61	15
			116	Fluorouracil and methotrexate	59	
0111	III	Breast	7	Anthracycline (every 3 weeks)	59	16
			6	Anthracycline (weekly)	50	
0204	rII	Non-small-cell lung	40	Docetaxel	66	19
0205	III	Colorectal	542	Fluorouracil and leucovorin	61	20
			536	UFT and leucovorin	61	
0207	III	Non-small-cell lung	62	Docetaxel	76	22
0407	rII	Gastric	49	Fluorouracil with or without methotrexate	59	29
			51	Paclitaxel	64	
0503	II	Ovarian	60	irinotecan and etoposide	58	30
0506	II	Pancreatic	50	Gemcitabine	67.5	32
0605	III	Small-cell lung	90	Nogitecan	64	35
0803	III	Non-small-cell lung	137	Docetaxel	76	37
0901	II	Small-cell lung	82	Amrubicin	66	39

Abbreviations: rII, randomized phase II.

### Incidence of platinum-based or nonplatinum-based chemotherapy-induced hyponatremia

**Figure 2:**
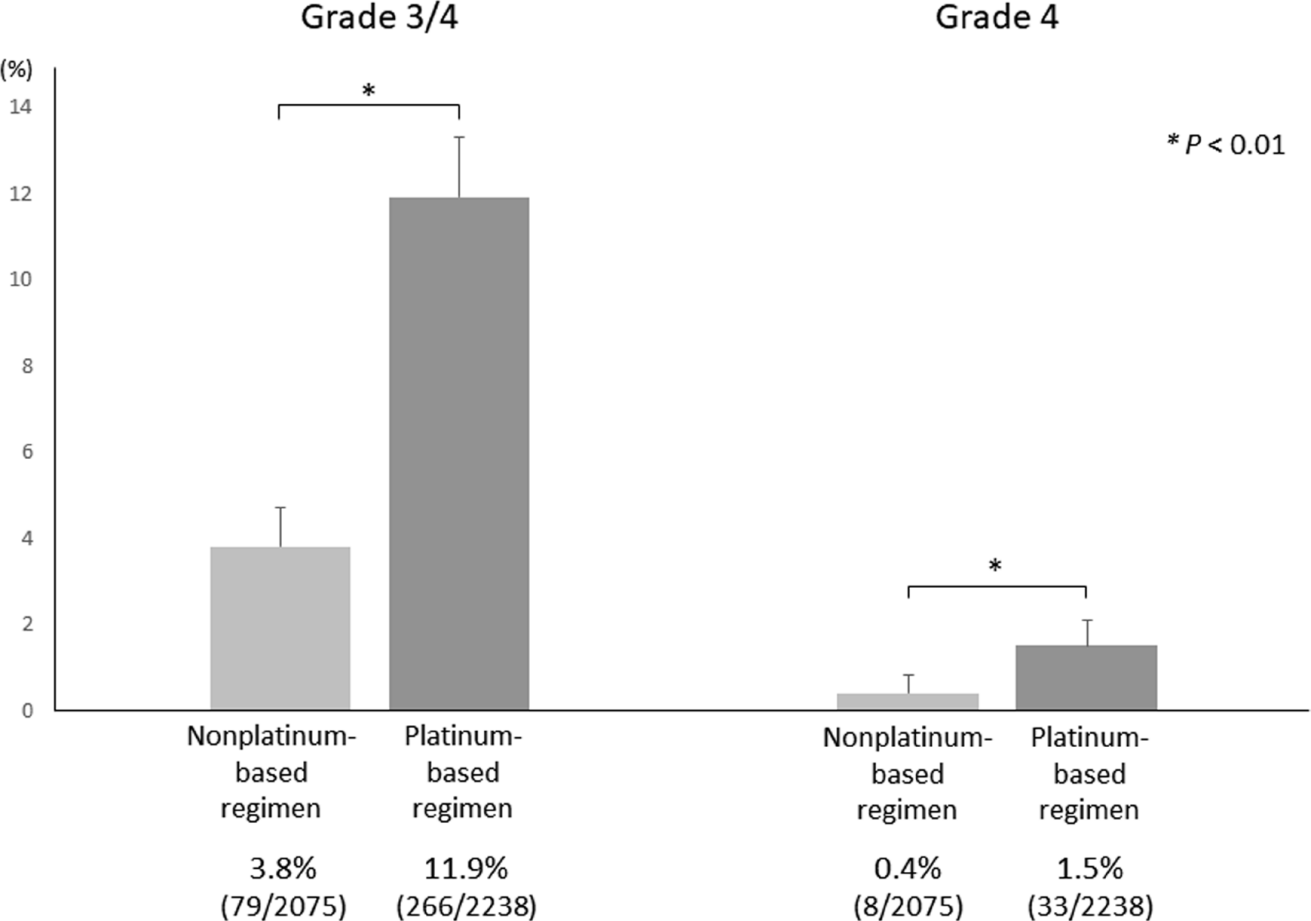
Incidence of chemotherapy-induced hyponatremia in patients who received platinum-based chemotherapy or nonplatinum-based chemotherapy

There was heterogeneity in the incidence of grade 3/4 hyponatremia among patients receiving platinum-based chemotherapy, ranging from 2.6% to 29.1% in the 27 included treatment arms (2,238 patients). The incidence of grade 3/4 hyponatremia in patients receiving platinum-based chemotherapy (11.9%) was significantly higher than that in patients receiving nonplatinum-based regimens (3.8%; *P* < 0.01). Similarly, the incidence of grade 4 hyponatremia in patients receiving platinum-based chemotherapy (1.5%) was significantly higher than that in patients receiving nonplatinum-based regimens (0.4%; *P* < 0.01) (Figure 2). All trials in this analysis reported no grade 5 hyponatremia and no serious aftereffects related to hyponatremia.

### The potential risk factors of platinum-induced hyponatremia

To evaluate the association between platinum and hyponatremia, we performed univariable analysis (Table 2).

**Table 2:** Subgroup analysis of incidence of platinum-based chemotherapy-induced hyponatremia

			Grade 3/4				Grade 4			
Subgroup	No. of treatment arms	No. of patients	No. of events	Incidence (%)	95% CI	*P* value	No. of events	Incidence (%)	95% CI	*P* value
**Type of platinum agent**										
Carboplatin	6	607	46	7.6	5.6–10.0	<0.01	2	0.3	0.0–1.2	<0.01
Cisplatin	21	1631	220	13.5	11.9–15.2		31	1.9	1.3–2.7	
**Administration methods**										
Split	8	606	69	11.4	9.0–14.2	0.71	6	1.0	0.4–2.1	0.32
Bolus	19	1632	197	12.1	10.5–13.8		27	1.7	1.1–2.4	
**Weekly equivalent cisplatin dose, mg/m**^2^										
<20	12	1037	127	12.2	10.3–14.4	0.06	14	1.4	0.7–2.3	0.04
≥20	9	594	93	15.7	12.8–18.8		17	2.9	1.7–4.5	
**Number of concomitant medications**										
0 (Platinum alone)	1	96	7	7.3	3.0–14.5	<0.01	0	0.0	0.0–3.8	0.03
1 (two-drug combination regimen)	22	1875	222	11.8	10.4–13.4		27	1.4	1.0–2.1	
2 (three-drug combination regimen)	2	145	31	21.4	15.0–29.0		6	4.1	1.5–8.8	
3 (four-drug combination regimen)	2	122	6	4.9	1.8–10.4		0	0.0	0.0–3.0	
**Types of concomitant medications**										
Vinorelbine	1	38	1	2.6	0.1–13.8	<0.01	0	0.0	0.0–9.3	<0.01
Taxane	9	875	82	9.4	7.5–11.5		6	0.7	0.3–1.5	
Fluorouracil	9	625	72	11.8	9.1–14.3		4	0.6	0.2–1.6	
Irinotecan	2	197	36	18.3	13.1–24.4		8	4.1	1.8–7.8	
Amrubicin	1	140	31	22.1	15.6–29.9		9	6.4	3.0–11.9	
**Tumor type**										
Bladder	1	56	3	5.4	1.1–14.9	<0.01	0	0.0	0.0–6.4	<0.01
Cervical	3	317	18	5.7	3.4–8.8		2	0.6	0.1–2.3	
Gastric	3	156	14	9.0	5.0–14.6		1	0.6	0.0–3.5	
Ovarian, fallopian tube and peritoneal	4	385	35	9.1	6.4–12.4		2	0.5	0.1–1.9	
Non-small-cell lung	5	373	40	10.7	7.8–14.3		2	0.5	0.1–1.9	
Esophageal/head and neck	8	579	82	14.2	11.4–17.3		7	1.2	0.5–2.5	
Small-cell lung	3	372	74	19.9	16.0–24.3		19	5.1	3.1–7.9	
**Median age**										
≥70 years old	3	295	33	11.2	10.6–13.5	0.77	2	0.7	0.1–2.4	0.30
<70 years old	24	1943	233	12.0	7.8–15.4		31	1.6	1.1–2.3	

Abbreviations: CI, confidence interval.

### Influence of the type of platinum agent

The incidence of grade 3/4 hyponatremia was greater in the cisplatin arm (13.5%) than the carboplatin arm (7.6%). The difference between these subgroups was statistically significant (*P* < 0.01). A similar tendency was observed in the analysis of grade 4 hyponatremia (1.9% in the cisplatin arm vs 0.3% in the carboplatin arm; *P* < 0.01).

### Influence of the platinum agent administration method

The incidence of grade 3/4 hyponatremia in the bolus arm was 12.1%, and that in the split arm was 11.4%; there was no significant difference between these subgroups (*P* = 0.71). A similar tendency was observed in the analysis of grade 4 hyponatremia (1.7% in the bolus arm vs 1.0% in the split arm; *P* = 0.32).

### Influence of cisplatin dosage

The incidence of grade 3/4 hyponatremia was greater in the high dosage arm (15.7%) than the low dosage arm (12.2%); however, there was no significant difference between these subgroups (*P* = 0.06). The incidence of grade 4 hyponatremia was significantly greater with high dosage (2.9%) than low dosage (1.4%; *P* = 0.04).

### Influence of the number of concomitant medications

**Figure 3:**
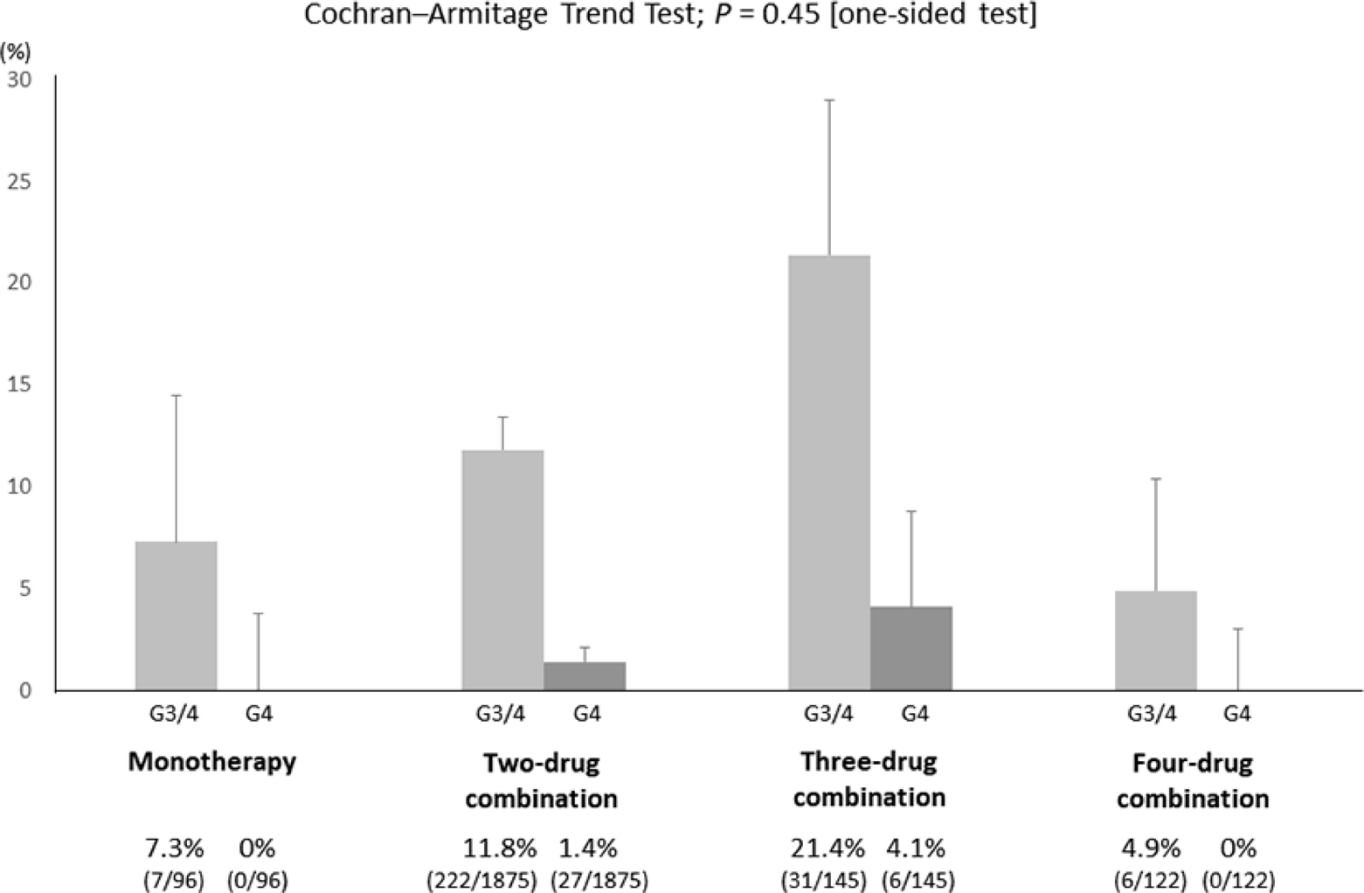
Incidence of platinum-induced hyponatremia in patients who received a platinum agent in monotherapy, two-drug, three-drug, and four-drug combination regimens

The incidence of grade 3/4 hyponatremia was highest in the three-drug combination regimen arm (21.4%), followed by the two-drug combination regimen (11.8%), monotherapy (7.3%), and four-drug combination (4.9%). The differences among these subgroups were significant (*P* < 0.01); however, the incidence of hyponatremia in the four-drug combination was lowest among four subgroups, and a Cochran–Armitage trend test did not show statistical significance (*P* = .45; one-sided test) (Figure 3). The incidence of grade 4 hyponatremia showed a similar tendency to that of grade 3/4 hyponatremia.

### Influence of the type of concomitant medication

The incidence of grade 3/4 hyponatremia was highest in the amrubicin combination regimen (22.1%), followed by irinotecan (18.3%), fluorouracil (11.8%), taxane (9.4%), and vinorelbine (2.6%). The differences among these subgroups were significant (*P* < 0.01). The incidence of grade 4 hyponatremia showed a similar tendency to that of grade 3/4 hyponatremia (*P* < 0.01).

### Influence of underlying tumor type

The incidence of grade 3/4 hyponatremia was highest among patients with small-cell lung cancer (19.9%), followed by esophageal/head and neck cancer (14.2%), non-small-cell lung cancer (10.7%), ovarian, fallopian tube and peritoneal cancer (9.1%), gastric cancer (9.0%), cervical cancer (5.7%), and bladder cancer (5.4%). The differences among tumor types were statistically significant (*P* < 0.01). The incidence of grade 4 hyponatremia was relatively higher in small-cell lung cancer (5.1%) than in the other cancers (*P* < 0.01).

### Influence of patients’ age

The incidences of grade 3/4 hyponatremia in elderly and nonelderly patients were 11.2% and 12.0%, respectively. There was no significant difference between these subgroups (*P* = 0.77). The incidence of grade 4 hyponatremia showed a similar tendency to that of grade 3/4 hyponatremia (*P* = 0.30).

## DISCUSSION

We conducted a cross-sectional, descriptive epidemiological study using data from completed clinical trials undertaken by JCOG in order to investigate the incidence of platinum-induced hyponatremia and its associated potential risk factors. The study design provided the following advantages. (a) By using data from patients who were enrolled into clinical trials without major deficiency of organ function, most cases of hyponatremia with causes other than the administration of platinum agents could be excluded. (b) We could obtain an adequate sample size; moreover, hyponatremia related to various administration methods and patient backgrounds enabled us to investigate potential risk factors. (c) Because JCOG trials are managed under JCOG policies by means of periodic data submission and quality control of the collected data, we could use good-quality data without missing values. Based on these advantages, we believe that the results of this study are quite reliable.

Overall, grade 3/4 hyponatremia developed in 11.9% of patients who received platinum agents. This proportion was significantly higher than that observed in patients who did not receive platinum agents (3.8%), thereby supporting the hypothesis that platinum agents increase the risk of hyponatremia. However, among the studies that administered platinum agents, the incidence of platinum-induced hyponatremia varied greatly from 2.9% to 29.1%. Therefore, it follows that treatment with platinum agents should be administered with care.

No treatment-related deaths due to hyponatremia (grade 5) were observed in any of the trials included in this study. In each of the trials included, early detection and appropriate treatment of hyponatremia must have been provided, and they were effective in preventing grade 5 hyponatremia.

In our study, we performed univariable analysis to determine possible risk factors. The results indicated that factors associated with a high incidence of hyponatremia included cisplatin usage, a cisplatin administration dosage of ≥20 mg/m^2^/week, administration of three-drug combination regimen, administration of three-drug combination regimen with amrubicin or irinotecan, and small-cell lung cancer. In addition, because there were no differences in the incidence of hyponatremia associated with patient age or the platinum agent administration method, these factors may not be potential risk factors.

In terms of the type of platinum agent, the incidence of hyponatremia was significantly higher with cisplatin compared with carboplatin (13.5% vs 7.6%; *P* < 0.01); this could be attributed to the difference in renal toxicity profiles of both drugs. Because carboplatin is excreted via filtration from the renal glomerulus alone, with no involvement of renal tubules, it rarely causes tubular disorders. In contrast, after being excreted via filtration from the renal glomerulus, cisplatin is reabsorbed through the proximal tubule and transported into epithelial cells through the basal membrane from blood vessels around the interstitial tissue; therefore, cisplatin administration may cause tubular disorders [10, 40, 41]. Thus, the difference in the incidence of hyponatremia observed in this study might be attributed to differences in renal toxicity (tubular disorders). To avoid such renal toxicity, mass infusion or administration of diuretics is often provided when patients undergo chemotherapy with platinum agents. Nevertheless, such mass infusion or diuretics themselves can cause hyponatremia [4, 8, 9]. However, the results of our study did not reveal any significant differences in the incidence of hyponatremia between the bolus administration group that received mass infusion along with diuretics and the split administration group where these were not administered together (12.1% vs 11.4%; *P* = 0.71); therefore, such factors seem to have limited effects. In addition, anorexia often occurs with administration of platinum agents, and it can cause hyponatremia. However, findings from the trials included that reported anorexia demonstrated no significant correlation between grade 3/4 anorexia and grade 3/4 hyponatremia using Pearson’s correlation analysis (*P* = 0.46; data not shown), suggesting that anorexia had a limited influence on hyponatremia. Overall, and as previously reported, the primary pathophysiology of platinum-induced hyponatremia may be renal salt-wasting syndrome and SIADH (syndrome of inappropriate antidiuretic hormone secretion).

Regarding the association between the number of concomitant medications and hyponatremia, the incidence of hyponatremia increased as the number of concomitant medications increased from the monotherapy group to the three-drug combination regimen group. However, unexpectedly, the lowest incidence of hyponatremia was observed with the four-drug combination regimen. Trials utilizing the four-drug combination regimen include the JCOG0102 trial [13], targeting cervical cancer, and the JCOG0209 trial [23], targeting bladder cancer; the JCOG0102 trial involved administration of cisplatin at 14 mg/m^2^ on days 1–5, every 3 weeks for 6 weeks, and the JCOG0209 trial administered 70 mg/m^2^ on day 2, every 4 weeks for 8 weeks, indicating that dose intensity was not particularly low and there were no special conditions. The reason for the lowest incidence with the four-drug combination regimens remains unclear.

On investigating concomitant medications, our results indicated a specifically high incidence of hyponatremia for amrubicin and, by underlying tumor type, small-cell lung cancer. However, because amrubicin was only administrated in cases of small-cell lung cancer among the included trials in this study, we could not elucidate which of these two was the stronger influencing factor. The incidence of hyponatremia was 15.9% (13/82) in a trial where amrubicin was administered alone (JCOG0901) [39], which is extremely high compared with that of other trials in the nonplatinum-based chemotherapy group (3.3%; 66/1993). This might suggest that amrubicin itself has a marked effect on hyponatremia.

The primary limitation of this study was the fact that it was a descriptive epidemiological study that did not use individual raw data. Therefore, we were unable to analyze factors, such as the timing of hyponatremia onset, detailed profiles of patients in whom hyponatremia occurred, and any possible gender differences, which warrants an investigation of individual raw data. However, no treatment-related deaths or severe aftereffects due to hyponatremia, such as central pontine myelinolysis, were observed in any of the trials included in our study. Consequently, because our findings can be considered sufficiently valuable, as they demonstrate incidence and potential risk factors of platinum-induced hyponatremia, we did not pursue a more detailed investigation using individual raw data.

In conclusion, this study is the first report to demonstrate the actual incidence of platinum-induced hyponatremia and its associated potential risk factors using a large-scale sample size and highly reliable data. These results will be useful for early detection and appropriate treatment of platinum-induced hyponatremia. Careful monitoring of serum sodium level is needed when platinum is administered. We anticipate that these results will be used to not only improve the safety of platinum-based chemotherapy, but also as a useful reference for future medical research.

## MATERIALS AND METHODS

### Study selection

JCOG clinical trials that met the following criteria were included in this analysis: (1) the study protocol had been approved by the protocol review committee of JCOG after January 2000; (2) patient enrolments had finished before January 2014; (3) phase II or III trials of patients with solid cancer who had received systemic chemotherapy; and (4) data on hyponatremia were available. Data from unpublished trials were excluded if the agreement to use the data was withheld by the representative of each trial.

### Data source

Data about the incidence of grade 3/4 hyponatremia used for our analysis were evaluated using the National Cancer Institute-Common Toxicity Criteria (NCI-CTC) v. 2.0 or Common Terminology Criteria for Adverse Events (CTCAE) v. 3.0 or v. 4.0 as described in the most recent reports of included trials, the data of which are stored in the JCOG Data Center. In NCI-CTC v. 2.0 or CTCAE v. 3.0 and v. 4.0, grade 3 hyponatremia is defined as serum sodium concentration 120–<130 mmol/L and grade 4 as <120 mmol/L. In addition, from information provided in the trial reports, we confirmed the presence or absence of grade 5 hyponatremia, defined as a fatality, and any serious aftereffects related to hyponatremia. Data of individual patients stored in the database were not used in this study.

### Statistical analysis

The incidence of grade 3/4 hyponatremia was compared between a platinum-based chemotherapy arm and nonplatinum-based chemotherapy arm. To explore the risk factors of platinum-induced hyponatremia, included trials of patients receiving platinum-based chemotherapy were divided into more than one subgroup according to the following criteria: type of platinum agent (cisplatin vs carboplatin), administration method of the platinum agent (bolus vs split), cisplatin dosage (<20 mg/m^2^/week vs ≥20 mg/m^2^/week), number of concomitant medications (platinum monotherapy alone, two-, three-, or four-drug combination regimens), type of concomitant medications (vinorelbine, taxane, fluorouracil, irinotecan, or amrubicin) in a two-drug combination regimen, underlying tumor type (bladder, cervical, gastric, ovarian, fallopian tube and peritoneal, non-small-cell lung, esophageal/head and neck, or small-cell lung), and patients’ median age (<70 years old vs ≥70 years old). With regards to cisplatin, because various dosages and schedules were studied in the included trials, an equivalent weekly dosage was calculated to standardize cisplatin dosage (e.g., patients receiving 80 mg/m^2^ once every 4 weeks or 20 mg/m^2^ once per week would both be in the 20 mg/m^2^/week subgroup). With regard to patients’ median age, because three of 27 trials were studied with elderly patients, median age was categorized as a nonelderly subgroup (<70 years old) or elderly subgroup (≥70 years old). The incidence of grade 3/4 hyponatremia along with 95% confidence interval (CI) by Clopper and Pearson method was calculated for each subgroup. Fisher’s exact test was used to compare the incidence of hyponatremia among subgroups. A two-sided *P* value < 0.05 was considered statistically significant. Because all evaluations in this study are considered to be exploratory data analyses, multiplicity adjustment was not applied. Statistical analysis was performed using SAS software (v. 9.2+; SAS Institute, Cary, NC, USA).

## Abbreviations

JCOG: Japan Clinical Oncology Group
SIADH: syndrome of inappropriate antidiuretic hormone secretion
NCI-CTC: National Cancer Institute-Common Toxicity Criteria
CI: confidence interval
